# Optimal therapeutic adropin dose intervention in mice and rat animal models: A systematic review

**DOI:** 10.14202/vetworld.2021.1426-1429

**Published:** 2021-06-04

**Authors:** Foad Alzoughool, Mohammad Borhan Al-Zghoul

**Affiliations:** 1 Department of Medical Laboratory Sciences, Faculty of Applied Medical Sciences, The Hashemite University, Zarqa, Jordan; 2 Basic Veterinary Sciences, School of Veterinary Medicine, Jordan University of Science and Technology, Irbid, Jordan

**Keywords:** adropin, animal model, dyslipidemia, glucose homeostasis, systematic review, therapeutic dose

## Abstract

**Background and Aim:**

Adropin is a hormone encoded by the *Enho* gene, which is associated with energy homeostasis. Preclinical studies using animal models have shown that adropin plays a role in enhancing glucose homeostasis and dyslipidemia. Lately, several studies on animal models have been performed to examine the therapeutic and pathophysiological effects of adropin in many disorders. The aim of this systematic review was to identify the ideal adropin dose in mice and rat animal models.

**Materials and Methods:**

We systematically searched PubMed, Science Direct, and Scopus databases from 2008 to 2020. The terms used in the search were “adropin,” “adropin doses in animal models,” “glucose homeostasis related to adropin,” and “adropin therapeutic effects on rats and mice.” Articles that included non-adropin doses, *in vitro* studies, and factors affecting adropin levels were excluded from the study.

**Results:**

Of the total 179 qualified studies, six studies were included. We found that a daily injection of 450 nmol/kg of adropin for 3 days might be considered the optimum dose of effect in mice, whereas injection of 2.1 mg/kg once a day for 10 successive days might be the optimal effective dose in rats.

**Conclusion:**

Additional investigations are needed to determine the optimum dose of adropin to be used as a therapeutic intervention depending on the animal model.

## Introduction

The new peptide hormone adropin, which is composed of 76 amino acids highly expressed in the liver, brain, and plasma, is encoded by the *Enho* gene, which is highly conserved in mammals and involved in energy homeostasis and lipid metabolism [1]. Adropin levels were found to be significantly higher in mice fed a high-fat, low-carbohydrate diet as compared with those fed a low-fat, high-carbohydrate diet, suggesting an important role of adropin in metabolic homeostasis [2]. The normal expression level of human plasma adropin is within the range of 1-10 mg/L, with a moderately higher expression in males than in females, and this level declines with age [3]. Adropin can be considered an important component in the pathophysiological pathways of several diseases, such as cardiovascular diseases, diabetes mellitus, endothelial dysfunction, hypertension, and chronic kidney disease [4-7].

In adropin knockout mice, a deficiency of adropin was found to be associated with hepatosteatosis and insulin resistance [2]. On the other hand, adropin improves cardiac energy metabolism in obese, pre-diabetic mice by increasing cardiac glucose oxidation under high-fat-diet conditions [8]. Adropin was also reported to enhance lipid metabolism, decrease insulin resistance, and suppress the inflammation of hepatocytes [9]. It was also observed that adropin treatment could improve glucose homeostasis through the suppression of peroxisome proliferator-activated receptor-gamma coactivator-1a, which regulates the expression of *Cpt1b*, *Cd36*, and *Pdk4* genes [10]. These data support the theory that adropin may be a promising drug target in the development of treatments against several diseases, such as cardiovascular diseases, diabetes mellitus, obesity, hypertension, and chronic kidney disease.

Several studies [2,8,10] have investigated the effect of interventional adropin in several animal models. The aim of this systematic review was to identify the ideal adropin dose in mice and rat animal models and to assist in future experimental studies on the decrease in adropin consumption in a cost-effective manner.

## Materials and Methods

### Ethical approval

This is a systematic review and it does not need ethical approval.

### Study selection

**Figure-1:**
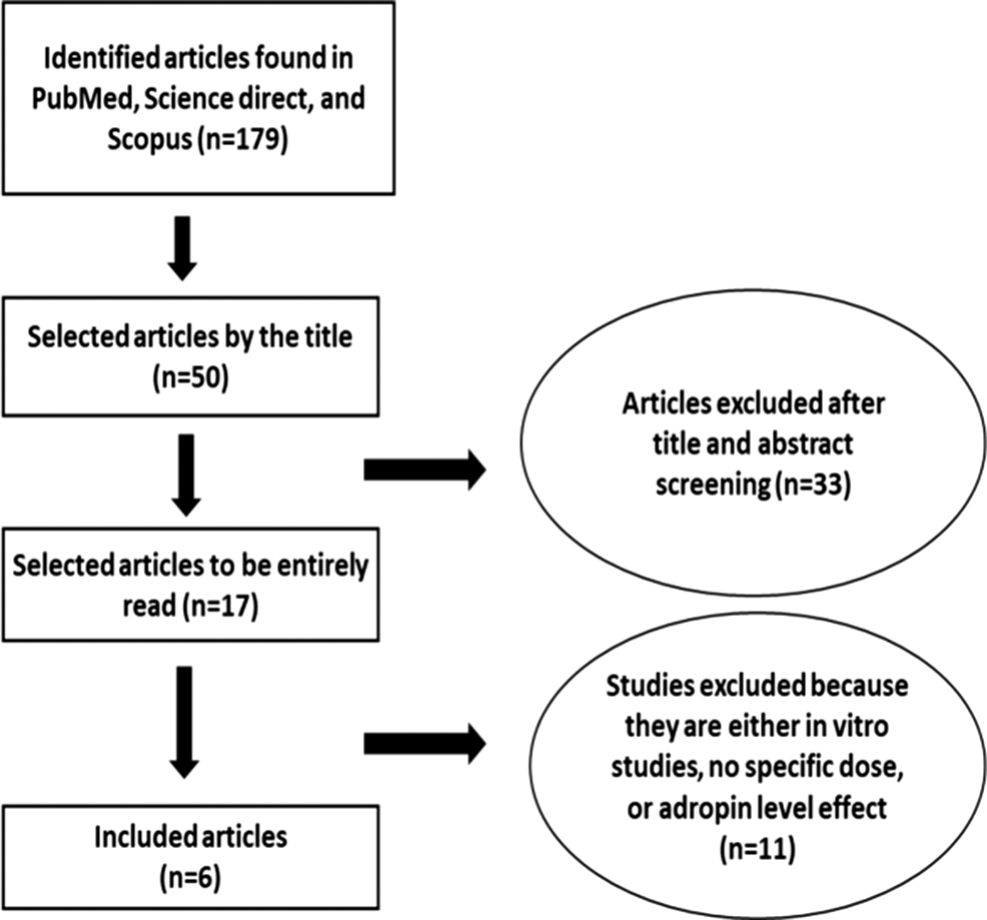
Inclusion criteria of systematic review.

We conducted a strong systematic search using the global web databases PubMed, Science Direct, and Scopus to determine the optimal dose of adropin in mice and rat animal models. To understand the mechanism of this myokine, we began by looking for adropin in general, followed by a hand search of abstracts of adropin and energy metabolism, especially in animal models (rats and mice). All selected abstracts were from the year 2008 until the present. In detail, we selected studies obtained through searching for the following keywords: “Adropin,” “adropin doses in animal models,” “glucose homeostasis related to adropin,” and “adropin therapeutic effects on rats and mice.” We identified 179 studies sorted by best match in PubMed and 119 studies in Science Direct and Springer sorted by most recent. We excluded articles that used non-adropin doses, *in vitro* studies, and factors affecting adropin level, as shown in Figure-1.

Studies providing data about specific therapeutic adropin doses and routes, *in vivo*, and well-defined studies describing all diseases that therapeutically required adropin were included in the study. We excluded *in vitro* and investigational studies explaining the factors affecting adropin levels. Furthermore, only dose-dependent adropin studies that provided relevant data about the therapeutic dose of adropin were included in the study. Table-1 shows the inclusion criteria used for study selection.

**Table-1:** Inclusion criteria of systematic review.

Inclusion criteria
All included studies in this systematic review depend on the following;
• Effect of adropin in rats and mice animal models
• Insulin resistance-related adropin.
• Therapeutic adropin for specific disorders.
• Adropin dose on *Enco* gene expression.
• Studies from 2008 until the present.

### Data extraction

The following data were extracted and presented in a table: Author(s), year, (dose, time, and unit), mouse and rat type, adropin-producing company, and study aims. We collected data independently from six studies using a standardized criterion. All selected studies contained detailed information for the requested data.

## Results

### Study selection

The literature review process performed in this study is summarized and presented in the flow chart in Figure-1. In the final step, six eligible studies were included in this review and assessed by the inclusion and exclusion criteria.

### Study characteristics

Overall, 6 of 179 studies were included in this systematic review. All included studies were published between 2008 and 2020, because adropin was discovered in 2008. The included studies were carried out on male and female rats and mice. Four studies were conducted using male C57BL/6 mice [8,10-12], and two studies used female Wistar albino rats [9,13]. The studies that used female Wistar albino rats evaluated the exposure of interventional adropin once a day for 10 successive days [9,13]. On the other hand, the studies that used male C57BL/6 mice examined the exposure of interventional adropin in five serial intraperitoneal injections over 3 days [8,10-12], as shown in Table-2. Adropin used in these experiments was obtained from different production companies: Phoenix Pharmaceuticals (Burlingame, CA, USA) [9,13], ChinaPeptides (Shanghai, China) [10,12], Ipsen (Paris, France) [10], and the University of Pittsburgh Peptide and Peptoid Synthesis Core (Pittsburgh, PA, USA) [8,11]. All included studies used intraperitoneal injections. Table-2 presents a summary of the studies.

**Table-2:** Studies summarization.

Study author and year	Type of injection	Aims	Company produced adropin	Mice type and info	Significant dose μg/kg	Dose frequency
Thapa *et al.*, 2019 [11]	Intraperitoneal injections	To examine the role of adropin in glucose homeostasis *in vivo*	Synthesized by the University of Pittsburgh Peptide and Peptide Synthesis Core	Male C57BL/6	450 nmol/kg	For 3 days
Gao *et al.*, 2019 [12]	Intraperitoneal injections	To investigate the adropin actions on ER stress and JNK activity To explore the effect of adropin on cAMP-dependent signaling pathways in the liver	China Peptides (Shanghai, China)	Male DIO B6 mice C57BL/6	450 nmole/kg	Five intraperitoneal
Thapa *et al.*, 2019 [8]	Intraperitoneal injections	To investigate if adropin role in restoration of glucose oxidation in the hearts of pre-diabetic obese mice	Synthesized by the University of Pittsburgh Peptide and Peptide Synthesis Core	Male C57BL/6	450 nmol/kg	Five serial intraperitoneal injections over 3 days
Akcılar *et al.*, 2015 [9]	Intraperitoneal injections	To investigate the possible alterations in blood glucose and lipid metabolism in response to adropin in hyperlipidemic and healthy rats.	Phoenix Pharmaceuticals, ABD	Wistar albino female rat (10 weeks old)	2.1 μg/kg/day	Once a day for continuous 10 days
Gao *et al.*, 2015 [10]	Intraperitoneal injections	To investigate whether adropin treatment would impact substrate utilization, improve glucose homeostasis, and ameliorate insulin resistance in the diet-induced obesity.	Ipsen (Paris, France) or China Peptides (Shanghai, wChina)	DIO C57BL/6 mice	450 nmole/kg	Five serial intraperitoneal injections
Akcılar *et al.*, 2016 [13]	Intraperitoneal injections	To examine the effects of adropin on glucose and lipid metabolism in a rat model of Type 2 diabetes mellitus	Phoenix Pharmaceuticals, ABD	Female Wistar albino rat weighing 250-300 g	2.1 μg/kg/day	Once a day for continuous 10 days

DIO=Diet-induced obese

## Discussion

This systematic review aimed to determine the optimum dose of adropin by clarifying the doses used in animal models. The following paragraphs describe the doses of adropin used in different animal pathophysiological models.

Several experimental *in vivo* studies investigated the effect of adropin interventions in glucose homeostasis. The findings of these investigations showed that intraperitoneal administration of adropin reduced fasting blood glucose levels in mice [11,12] and rats [9,13]. Treatment of obese mice with a single dose of 450 nmol/kg adropin for 3 days decreased the hepatic glucose production in adropin-treated mice fed a high-fat diet as compared with controls [11]. In a study performed by Gao *et al*. [12], five adropin intraperitoneal injections (450 nmol/kg) in ­diet-induced obese (DIO) male mice enhanced glucose homeostasis through mediation of the hepatic regulation of glucose metabolism [12]. In rats, intraperitoneal injection of adropin (2.1 mg/kg/day) for 10 days was associated with a significant reduction in blood glucose levels [9,13]. These data suggest that adropin might be used to treat glycemia.

In addition to its role in glucose reduction in mice and rats, adropin intervention lowered the serum levels of total cholesterol, triglycerides, low-density lipoprotein cholesterol levels, and increased high-density lipoprotein cholesterol levels [9,13], suggesting a therapeutic effect of adropin on hyperlipidemia. Adropin was found to improve liver enzyme markers involved in hyperlipidemia, including aspartate transaminase (AST), alanine transaminase (ALT), and gamma-glutamyl transferase (GGT) [13].

Adropin was reported to enhance glucose and insulin tolerance in DIO mice through insulin intracellular signaling through the Akt pathway in muscles. Adropin was suggested to induce sensitization of the insulin signaling pathways by increasing the expression of GLUT4 on the cell surface and inducing phosphorylation of Akt [10]. The increased surface expression of GLUT4 in response to insulin suggests a potential increase in muscle glucose uptake. The study showed that adropin treatment appears to sensitize the Akt response to insulin by downregulating phosphatase and tensin homolog, with a possible increase in the basal level of PIP3 [10]. Moreover, by reducing c-Jun N-terminal kinase (JNK) activity in the liver, adropin interventions suppressed hepatic glucose production and improved hepatic insulin sensitivity [12]. It is well known that JNK antagonizes insulin resistance by regulating the expression of pro-inflammatory cytokines [14]. Adropin was also reported to significantly enhance the alterations in tumor necrosis factor-α, interleukin-6, and inducible nitric oxide synthase mRNA expression [9].

In the high-fat-diet mice model, five serial intraperitoneal injections of adropin (450 nmol/kg) were administered over 3 days. It was found that adropin adjusts the cardiac energy metabolism in obese mice [8]. Treatment with adropin significantly reversed the decreased activity of cardiac pyruvate dehydrogenase that was shown in high-fat mice [8].

All studies included in our systematic review provide robust evidence on the efficiency of adropin intervention as a therapeutic agent for several pathophysiological conditions that affect glucose homeostasis and lipid metabolism in several types of ­animal models. Adropin intervention was reported to prevent hyperglycemia and hyperlipidemia; improve glucose tolerance; increase whole-body insulin sensitivity; inhibit hepatic glucose production; ameliorate hepatic insulin sensitivity; decrease cardiac glucose oxidation and cardiac pyruvate dehydrogenase activity; reduce serum levels of total cholesterol, triglycerides, low-density lipoprotein cholesterol, AST, ALT, alkaline phosphatase, and GGT; and increase the level of high-density lipoprotein cholesterol. In addition, adropin intervention downregulated peroxisome proliferator-activated receptor-gamma coactivator-1a, which regulates the expression of Cpt1b, Cd36, and Pdk4.

In the studies using mice as an animal model, the optimal dose of adropin showing a therapeutic effect was 450 nmol/kg [8,11,12]. On the other hand, the optimal adropin dose showing a therapeutic effect in studies using rats as an animal model was 2.1 mg/kg once a day for 10 successive days [9,13].

## Conclusion

In the present review, we summarized all potential adropin intervention doses used in mice and rat models between 2008 and 2020. The optimal dose of adropin in mice, especially C57BL/6 mice, was 450 nmol/kg daily, whereas in rats, especially Wistar albino rats, it was 2.1 mg/kg/day. Additional investigations are needed to determine the optimal dose of adropin to be used as a therapeutic intervention depending on the animal model.

## Authors’ Contributions

FA and MBA: Collected and analyzed the data, formal analysis, funding acquisition, original draft preparation, reviewed and edited the manuscript. Both authors read and approved the final and revised copy of the manuscript.

## References

[1] Kumar K.G, Trevaskis J.L, Lam D.D, Sutton G.M, Koza R.A, Chouljenko V.N, Kousoulas K.G, Rogers P.M, Kesterson R.A, Thearle M, Ferrante A.W, Mynatt R.L, Burris T.P, Dong J.Z, Halem H.A, Culler M.D, Heisler L.K, Stephens J.M, Butler A.A (2008). Identification of adropin as a secreted factor linking dietary macronutrient intake with energy homeostasis and lipid metabolism. Cell Metab.

[2] Kumar K.G, Zhang J, Gao S, Rossi J, McGuinness O.P, Halem H.H, Culler M.D, Mynatt R.L, Butler A.A (2012). Adropin deficiency is associated with increased adiposity and insulin resistance. Obesity.

[3] Zhang S, Chen Q, Lin X, Chen M, Liu Q (2020). A review of adropin as the medium of dialogue between energy regulation and immune regulation. Oxid. Med. Cell Longev.

[4] Hu W, Chen L (2016). Association of serum adropin concentrations with diabetic nephropathy. Mediators Inflamm.

[5] Aydin S, Kuloglu T, Aydin S, Kalayci M, Yilmaz M, Çakmak T, Eren M.N (2014). Elevated adropin:A candidate diagnostic marker for myocardial infarction in conjunction with troponin-I. Peptides.

[6] Lin D, Yong J, Ni S, Ou W, Tan X (2019). Negative association between serum adropin and hypertensive disorders complicating pregnancy. Hypertens. Pregnancy.

[7] Shelest B.B, Kovaleva Y.I.V, Rodionova I.V (2019). Adropin as a prognostic factor in heart failure development in hypertensive patients. Eur. J. Heart Fail.

[8] Thapa D, Xie B, Zhang M, Stoner M.W, Manning J.R, Huckestein B.R, Edmunds L.R, Mullett S.J, McTiernan C.F, Wendell S.G, Jurczak M.J, Scott I (2019). Adropin treatment restores cardiac glucose oxidation in pre-diabetic obese mice. J. Mol. Cell Cardiol.

[9] Akcilar R, Kocak F.E, Simsek H, Akcilar A, Bayat Z, Ece E, Kokdasgil H (2016). Antidiabetic and hypolipidemic effects of adropin in streptozotocin-induced type 2 diabetic rats. Bratisl. Lek. Listy.

[10] Gao S, McMillan R.P, Zhu Q, Lopaschuk G.D, Hulver M.W, Butler A.A (2015). Therapeutic effects of adropin on glucose tolerance and substrate utilization in diet-induced obese mice with insulin resistance. Mol. Metab.

[11] Thapa D, Xie B, Manning J.R, Zhang M, Stoner M.W, Huckestein B.R, Edmunds L.R, Zhang X, Dedousis N.L, O'Doherty R.M, Jurczak M.J, Scott I (2019). Adropin reduces blood glucose levels in mice by limiting hepatic glucose production. Physiol. Rep.

[12] Gao S, Ghoshal S, Zhang L, Stevens J.R, McCommis K.S, Finck B.N, Lopaschuk G.D, Butler A.A (2019). The peptide hormone adropin regulates signal transduction pathways controlling hepatic glucose metabolism in a mouse model of diet-induced obesity. J. Biol. Chem.

[13] Akcılar R, Koçak F.E, Şimşek H, Akcılar A, Bayat Z, Ece E, Kökdaşgil H (2016). The effect of adropin on lipid and glucose metabolism in rats with hyperlipidemia. Iran. J. Basic Med. Sci.

[14] Solinas G, Becattini B (2017). JNK at the crossroad of obesity, insulin resistance, and cell stress response. Mol. Metab.

